# Cytokine polarized, alternatively activated bone marrow neutrophils drive axon regeneration

**DOI:** 10.21203/rs.3.rs-3491540/v1

**Published:** 2023-10-31

**Authors:** Andrew D. Jerome, Andrew R. Sas, Yan Wang, Jing Wen, Jeffrey R. Atkinson, Amy Webb, Tom Liu, Benjamin M. Segal

**Affiliations:** Department of Neurology, The Ohio State University; The Neuroscience Research Institute, The Ohio State University; Department of Neurology, The Ohio State University; The Neuroscience Research Institute, The Ohio State University; Department of Neurology, The Ohio State University; The Neuroscience Research Institute, The Ohio State University; The James Comprehensive Cancer Center, The Ohio State University; Department of Neurology, The Ohio State University; The Neuroscience Research Institute, The Ohio State University; The James Comprehensive Cancer Center, The Ohio State University; Department of Biomedical Informatics, The Ohio State University; Department of Neurology, The Ohio State University; The Neuroscience Research Institute, The Ohio State University; Department of Neurology, The Ohio State University; The Neuroscience Research Institute, The Ohio State University

## Abstract

The adult central nervous system (CNS) possesses a limited capacity for self-repair. Severed CNS axons typically fail to regrow. There is an unmet need for treatments designed to enhance neuronal viability, facilitate axon regeneration, and ultimately restore lost neurological functions to individuals affected by traumatic CNS injury, multiple sclerosis, stroke, and other neurological disorders. Here we demonstrate that both mouse and human bone marrow (BM) neutrophils, when polarized with a combination of recombinant interleukin (IL)-4 and granulocyte-colony stimulating factor (G-CSF), upregulate alternative activation markers and produce an array of growth factors, thereby gaining the capacity to promote neurite outgrowth. Moreover, adoptive transfer of IL-4/G-CSF polarized BM neutrophils into experimental models of CNS injury triggered substantial axon regeneration within the optic nerve and spinal cord. These findings have far-reaching implications for the future development of autologous myeloid cell-based therapies that may bring us closer to effective solutions for reversing CNS damage.

Axonal transection and neuronal death are prevalent in neurological conditions caused by a wide range of insults, including physical trauma, autoimmune inflammation, ischemia-reperfusion, and neurodegenerative disease. The adult mammalian central nervous system (CNS) has a limited capacity for self-repair, rendering it particularly susceptible to injury. Unlike their counterparts in the developing CNS, mature neurons lack the ability to replicate, and severed axons typically do not regrow, thereby heightening the risk of unfavorable neurological outcomes. At present, there is a dearth of therapies that directly foster healing processes in the wake of CNS damage to reverse neurological deficits. This underscores a significant unmet need for treatments specifically designed to enhance neuronal viability, facilitate CNS axon regeneration, and ultimately restore lost neurological functions.

Traditionally, neuroinflammation has been perceived to be detrimental in the context of CNS damage and repair. Immune cells, mobilized in response to trauma, infection, or other insults, could cause collateral damage to healthy tissues by releasing harmful substances such as free oxygen radicals, enzymes and pro-apoptotic factors. However, it is now widely understood that, during later stages of inflammation, alternative pathways emerge within the innate immune system that contribute to tissue repair and restoration of homeostasis. Monocytes and monocyte-derived cells, expressing markers of “alternative activation” such as arginase-1 (Arg1) and mannose receptor (Mrc1, CD206), drive healing processes in animal models of cutaneous wounds, atherosclerosis, and myocardial ischemia^1–5^. Growing evidence also supports the existence of immune-mediated healing within the CNS^6^. The precise identity of CNS-infiltrating reparative leukocytes, the characteristics that distinguish them from pathogenic subsets, and the mechanisms underlying their actions, remain elusive.

Recent advances in high-dimensional technologies have shed light on the previously unrecognized heterogeneity, plasticity, and functional sub-specialization of neutrophils^7–9^. Studies employing such technologies have revealed a role of unconventional neutrophil subsets in animal models of stroke and myocardial infarction^10,11^. Notably, we recently identified a new subset of immature, alternatively activated Ly6G^low^ neutrophils that express Arg1, Mrc1 and IL-4 receptor α-chain (IL-4Rα), and possess neuroprotective and pro-regenerative properties^12,13^. These cells were initially discovered within the context of a well-established mouse model used for studying inflammation-driven CNS repair following optic nerve (ON) crush injury. Crush injury ordinarily results in extensive ON axon transection and loss of retinal ganglion cells (RGCs), the projection neurons of the eye. However, this damage can be mitigated, and partially reversed, by intraocular (i.o.) injection of zymosan, a fungal cell wall extract^13–16^, either at the time of trauma or up to 3 days afterwards. The i.o. zymosan treatment promotes robust RGC rescue and ON regeneration, associated with the accumulation of alternatively activated Ly6G^low^ neutrophils in the vitreal fluid. These atypical neutrophils secrete various growth factors, including nerve growth factor (NGF) and insulin-like growth factor-1 (IGF-1), which contribute to their pro-regenerative effects^12,13^. Harnessing the potential of pro-regenerative neutrophil lineage cells holds promise for innovative restorative therapies aimed at enhancing neuroprotection and re-establishing neuronal networks.

Immunotherapies involving the administration of zymosan, or zymosan-modulated cells, which might release zymosan particles post-administration, come with inherent limitations that need to be addressed before considering clinical translation. Potential complications include off target side effects of zymosan, as well as variations in the molecular composition and biological properties of the fungal cell wall extract between batches, posing challenges in ensuring consistent therapeutic results. The zymosan-modulated neutrophils that we investigated in our published research are enriched in alternatively activated Ly6G^low^ cells^13^. However, despite this enrichment, the collective cell population still exhibits a degree of heterogeneity^12^. To overcome this limitation, and enable deeper investigations into mechanisms of action, we sought to devise a protocol utilizing standardized polarizing reagents to reliably yield a more homogenous population of pro-regenerative myeloid cells.

Interleukin (IL)-4 is a critical factor in the development of alternatively activated macrophages^17^, while granulocyte-colony stimulating factor (G-CSF) promotes granulopoiesis. We found that short term culture of naive Ly6G^+^ murine bone marrow (BM) cells with a combination of recombinant mouse IL-4 and G-CSF reprograms those cells, causing them to upregulate alternative activation markers and produce an array of cytoprotective and growth factors, akin to their zymosan-modulated counterparts. Notably, these reprogrammed cells demonstrate a high degree of reproducibility in stimulating explanted RGC and dorsal root ganglion neurons to extend neurites. Moreover, when we adoptively transferred IL-4/G-CSF polarized BM neutrophils into experimental models of CNS injury, we observed favorable outcomes marked by significant axon regrowth within both the optic nerve and the spinal cord. Importantly, we discovered that IL-4/G-CSF polarized *human* bone marrow cells contain a subset of immature neutrophils capable of stimulating primary human cortical neurons to grow neurites. This discovery underscores the translational potential of our findings, bridging the gap between murine models and future clinical applications.

## Results

### IL-4/G-CSF polarized Ly6G^+^ BM cells display characteristics of alternatively activated, immature neutrophils.

**Figure 1:**
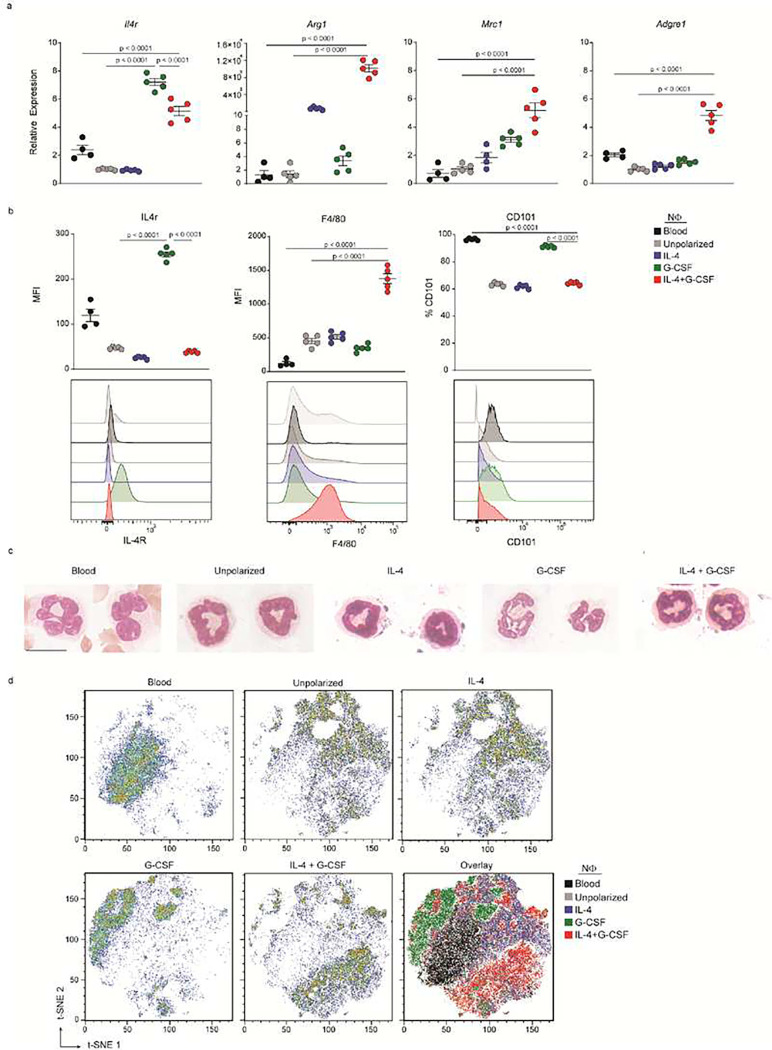
IL-4/G-CSF polarized murine bone marrow neutrophils (BMNΦ) exhibit an immature, alternatively activated phenotype. a-d, Ly6G+ neutrophils (NΦ) were isolated from naïve C57BL/6 bone marrow (BM) cells and cultured with or without polarizing factors for 24 hours. a, RNA was extracted from the cultured BMNΦ, or from peripheral blood NΦ directly ex vivo. Levels of transcripts encoding IL-4 receptor α chain (IL-4Rα, Il4r), arginase-1 (Arg1), mannose receptor (Mrc1), and F4/80 (Adgre1), were measured by RTqPCR and normalized to β-Actin (Actb). Data are presented as fold increase over mean normalized levels in unpolarized BM NΦ. Each symbol represents data from an individual mouse (n=4–5 mice per group). Data shown are from one experiment, representative of four independent experiments. b-c, Ly6G+ bone marrow cells cultured under the specified conditions, and Ly6G+ peripheral blood NΦ, were analyzed by flow cytometry. b, Cell surface expression of indicated molecules on Ly6G+ cells. Upper panels, mean fluorescence intensity (MFI) of cell surface IL4Rα and F4/80, and the percentage of CD101+ cells. Each symbol represents data from a single mouse. (n=4–5 mice per group). Lower panels display representative histograms. Data shown are representative of five independent experiments. c,Representative cytospins of Ly6G+ BM NΦ, after culture under the specified conditions, stained with Wright-Giemsa solution. Scale bar,10 μm. d, Representative sample and overlay t-SNE plots generated by combining flow cytometric data from the analysis of polarized and unpolarized BM NΦ, along with naïve peripheral blood NΦ. Data shown from one experiment, representative of four independent experiments. a, b, Statistical significance was determined using one-way ANOVA followed by Dunnett’s post hoc test

We isolated Ly6G^+^ cells from the BM of naïve C57BL/6 mice, and stimulated them with different cytokine cocktails in culture. We prioritized polarizing factors traditionally associated with alternative activation, including IL-4. The cultured cells were harvested after 24 hours and analyzed for expression of the alternative activation markers, IL-4Rα, Arg-1, and Mrc-1, by quantitative RT-qPCR (Fig. 1a), and flow cytometric analysis (Fig. 1b), respectively. Consistent with published reports from independent laboratories^18,19^, we found that naïve Ly6G^+^ BM cells express very low levels of IL-4Rα, directly *ex vivo*, or following short term culture under neutral conditions (Fig. 1a,b). However, they upregulated IL-4Rα, on both the mRNA and protein level, following direct stimulation with recombinant G-CSF. Ly6G^+^ BM cells cultured with a combination of recombinant G-CSF and IL-4 downregulated cell surface IL-4Rα compared with their counterparts that were cultured with G-CSF only, likely secondary to internalization of the IL-4 receptor upon binding IL-4 (Fig. 1b). The double cytokine stimulated cells expressed relatively high levels of *Arg1* and *Mrc1* mRNA (Fig. 1a). Irrespective of the culture conditions, Ly6G^+^ BM cells consistently expressed signature markers of the neutrophil lineage, such as myeloperoxidase and neutrophil elastase (Extended Data Fig. 1). However, only IL-4/G-CSF co-stimulated cells expressed high cell surface and transcript levels of F4/80 (*Adgre1*), a marker that is traditionally associated with macrophages (Fig. 1a,b). We concluded that G-CSF enhanced the responsiveness of immature granulocytes to IL-4, and subsequent IL-4 signaling induced alternative activation. IL-10 and TGFβ have been implicated in the induction of reparative immune cells^2^. However, Ly6G^+^ BM cells stimulated with IL-10 and TGFβ, either individually or together, did not upregulate either Arg-1, Mrc-1, IL-4rα or F4/80 (data not shown).

When Ly6G+ BM cells were stimulated with G-CSF alone, a substantial proportion of them expressed the maturation marker CD101, similar to the percentage seen in circulating neutrophils (Fig. 1b). In contrast, Ly6G^+^ cells that received co-stimulation with IL-4 and G-CSF exhibited a relatively low percentage of CD101^+^ cells. Ly6G^+^ BM cells acquired a segmented nuclear morphology following stimulation with G-CSF alone, but retained a ring shaped nucleus following stimulation with either IL-4 alone or with a combination of G-CSF and IL-4 (Fig. 1c). Collectively, these observations suggest that G-CSF driven maturation of Ly6G^+^ BM cells is suppressed by co-stimulation with IL-4.

We used t-distributed Stochastic Neighbour Embedding (t-SNE) to further analyze our multiparameter flow cytometry data, employing an expanded panel of markers consisting of CD45, CD11b, Ly6G, CD115, CD14, F4/80, Mrc1, IL-4Rα, and CD101. The majority of IL-4/G-CSF polarized Ly6G^+^ BM cells clustered together, consistent with their characterization as a distinctive subpopulation (Fig. 1d).

### IL-4/G-CSF polarized BM neutrophils (BMNΦ) promote RGC viability and stimulate ON axon regrowth.

**Figure 2:**
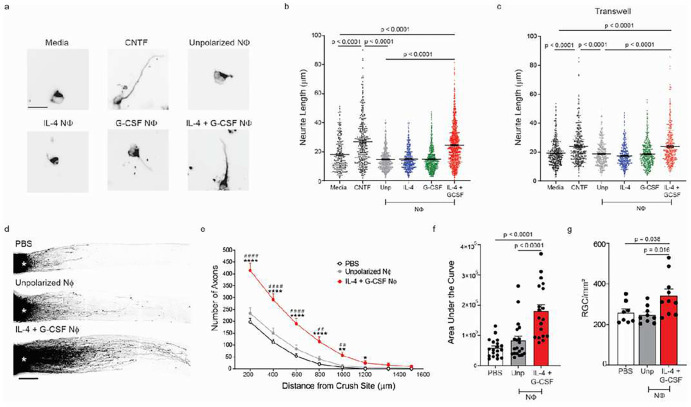
IL-4/G-CSF polarized bone marrow neutrophils enhance RGC survival and axon regrowth. a-c, Ly6G+ BMNΦ, polarized under the indicated conditions, were co-cultured with primary RGCs for 24 hours. RGCs and NΦ were either plated in direct contact with one another (a, b), or separated across a transwell (c). a, Representative images of RGCs from each group (scale bar = 20 μm). b, c, Mean length of the longest neurite grown by cultured RGCs. Each symbol represents one RGC. (n=3 mice per condition, > 400 RGCs per condition). RGCs were cultured with recombinant ciliary neurotrophic factor (CNTF) as a positive control, or media alone as a negative control (n > 150 RGCs per condition). Statistical significance was determined by one way ANOVA followed by Dunnett’s post hoc test. Data shown from one experiment, representative of three independent experiments. d-g, Mice were injected i.o. with IL-4/G-CSF polarized or unpolarized BMNΦ, or with PBS alone, on days 0 and 3 post-ONC injury. RGC uptake and anterograde transport of Alexa 647 conjugated CTB tracer was assessed on day 14 post-ONC. d, Representative images of optic nerves from each group. Scale bar, 200 μm. e, Density of CTB+ regenerating axons in longitudinal optic nerve sections, counted at serial distances from the crush site (n=18–20 nerves per group). f, Area under the curve (AUC) measured for each treatment group from the data shown in (e). Each symbol represents results obtained from an individual nerve (n=18–20 nerves per group). e, f, Data are representative of five independent experiments. g, Frequency of viable Brn3a+ RGCs per mm2 in retinal whole mounts (n=8–10 eyes per group). Each symbol represents results obtained from an individual retina. Data are from one experiment, representative of two independent experiments. e, Statistical significance was determined via unpaired t-test with Welch correction. *P < 0.05; **P < 0.01, ***P < 0.001 and ****P < 0.0001, compared with i.o. PBS; # P < 0.05, ##P < 0.01 and ####P < 0.0001, compared with unpolarized NΦ group. f, g, Statistical significance was determined by one-way ANOVA followed by Dunnett’s post hoc test

Next, we assessed the cytoprotective and pro-regenerative properties of IL-4/G-CSF polarized BMNΦ in comparison to their unpolarized, or single cytokine polarized, counterparts. Notably, only the BMNΦ polarized with both IL-4 and G-CSF exhibited the capacity to promote neurite outgrowth when co-cultured with explanted RGC *in vitro* (Fig. 2a,b). When administered i.o. on days 0 and 3 post-ONC injury, IL-4/G-CSF polarized, but not unpolarized, BMNΦ enhanced RGC viability and stimulated significant ON axon regeneration *in vivo* (Fig. 2c–f). Moreover, i.o. administration of the doubly polarized BMNΦ to mice with ONC injury did not exacerbate the reactive gliosis normally triggered by ON crush, as assessed by GFAP immunohistochemistry (Extended Data Fig. 2).

### IL-4/ G-CSF polarized BMNΦ express a distinctive transcriptome.

**Figure 3:**
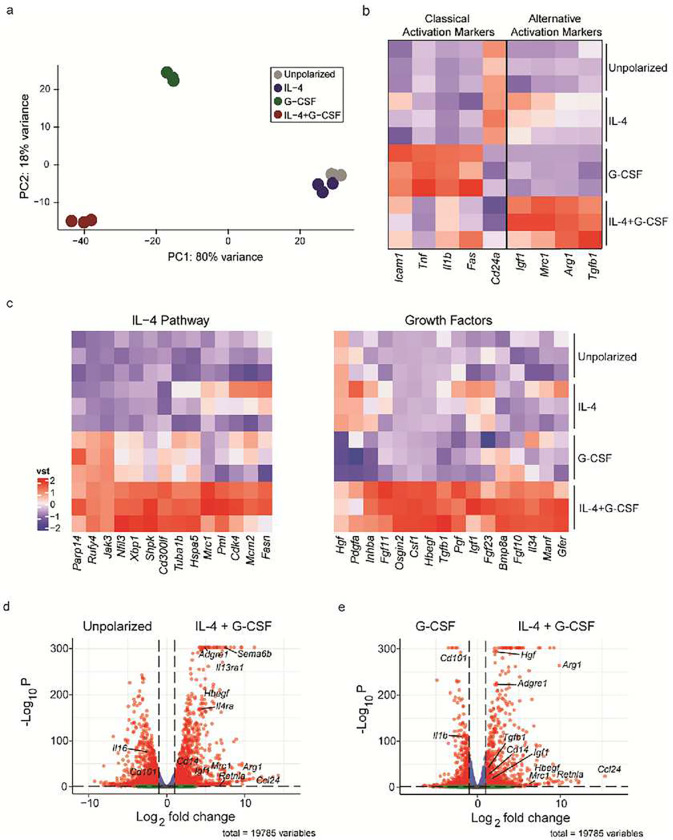
IL-4/G-CSF polarized neutrophils exhibit a transcriptome indicative of alternative activation and a regenerative phenotype. Purified Ly6G+ BMNΦ, harvested 24 hours after culture with or without the polarizing factors indicated, were analyzed by bulk RNA sequencing. a, Principal component analysis (PCA) plot. Each symbol represents data collected from one biological replicate (n=3). b, c Heat maps depicting scaled expression of selected genes associated with classical activation (b, left panel), alternative activation (b, right panel), or IL-4 signaling (c, left panel), or genes encoding growth factors (c, right panel). d, e, Volcano plots illustrating the differences in gene expression between unpolarized BMNΦ and IL-4/G-CSF polarized BMNΦ (d), and between G-CSF polarized BMNΦ and IL-4/G-CSF polarized BMNΦ (e). Red dots represent genes that are below an adjusted P value of 0.05 and greater than an absolute log2-fold change of 0.25. Green dots represent genes that meet the fold change threshold but not the P value threshold. Blue dots represent genes that meet the p value threshold, but not the fold change threshold

In order to characterize IL-4/G-CSF polarized BMNΦ in greater depth, and gain insight into their mechanism(s) of action, we performed bulk RNA sequencing of those cells in comparison to their unpolarized and single cytokine polarized counterparts. Principal Component Analysis (PCA) demonstrated that IL-4/G-CSF polarized BMNΦ readily separate from unpolarized and single cytokine polarized BMNΦ based on gene expression profiling (Fig. 3a). IL-4/G-CSF polarized BMNΦ expressed relatively high levels of genes associated with the IL-4 receptor signaling pathway, and genes that encode alternative activation markers and growth factors (Fig. 3b–e). The latter include IGF-1 (implicated in healing responses mediated by other innate immune cells, including zymosan-modulated neutrophils^13^) and heparin-binding epidermal growth factor-like growth factor (HB-EGF; implicated in neuroprotection, neurogenesis, and neuritogenesis^20–22^). In comparison to their IL-4/G-CSF or IL-4 polarized counterparts, G-CSF polarized BMNΦ differentially expressed genes associated with classical activation (Fig. 3b).

### *IL-4/G-CSF polarized* BMNΦ *produce neuroprotective and growth factors*.

**Figure 4:**
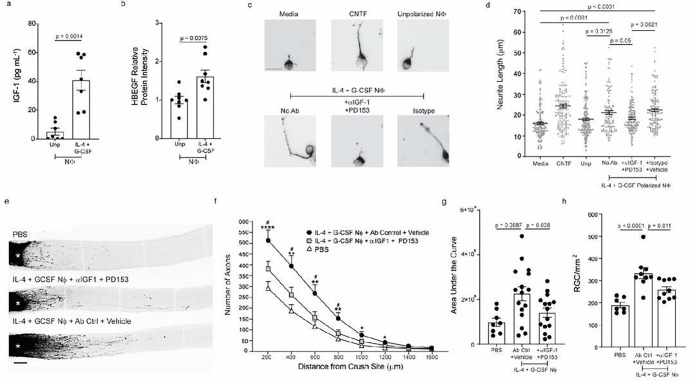
IL-4/G-CSF polarized BMNΦ enhance axon regeneration, in part, via the production of IGF-1 and HBEGF. a, b Ly6G+ BMNΦ were cultured either with recombinant IL-4 and G-CSF, or without recombinant cytokines (Unp), for 24 hours. a, Cells from both groups were harvested, washed, resuspended in fresh media, and cultured without exogenous cytokines. Conditioned media were collected 24 hours later. IGF-1 levels were measured via ELISA. b, HBEGF levels were measured in cell lysates via western blot analysis. Band intensity was normalized to total protein. Data are shown as fold increase over levels in unpolarized cells. a, b, Each dot represents one biological replicate. Pooled from 2 independent experiments. c, d, RGCs were co-cultured with IL-4/G-CSF polarized BMNΦ for 24 hours in the presence or absence of an anti-IGF-1 neutralizing antibody and/or a small molecule antagonist of the EGF receptor (PD153). c, Representative images of RGCs in each experimental group are displayed. Scale bar, 20 μm. d, Each symbol represents one RGC. Some RGCs were cultured with recombinant CNTF, as a positive control, while others were cultured in media alone, as a negative control. (n=3 mice per condition, > 400 RGCs per condition). Data shown are from one experiment, representative of two independent experiments. e-h, On days 0 and 3 post-ONC injury, mice received i.o. injections of PBS or IL-4/G-CSF polarized BMNΦ in combination with IGF-1 neutralizing antibody and PD153, or with control antibody and vehicle. Alexa 647-conjugated CTB tracer was injected i.o. on day 12 post injury. Optic nerves and retinas were harvested 2 days later. e, Representative images of CTB+ optic nerves. Scale bar, 200 μm. f, Density of CTB+ regenerating axons in longitudinal optic nerve sections at serial distances from the crush site (n=15–16 nerves per treatment group; n=8 nerves in PBS control group). Data shown are pooled from two independent experiments. g, Area under the curve (AUC) was measured using the data shown in (f). h, Frequency of viable Brn3a+ RGCs per mm2 in retinal whole mounts. (n=7–9 retinas per group). Data shown from one experiment, representative of two independent experiments. g, h, Each dot represents an individual nerve or retina. a, b, f, Statistical significance was determined by unpaired t-test with Welch correction. g, h, Statistical significance was determined by one-way ANOVA followed by Dunnett’s post hoc test. f, *P < 0.05; **P < 0.01, and ****P < 0.0001, compared with i.o. PBS; # P < 0.05, compared with with αIGF-1 + PD153 group

IL-4/G-CSF polarized BMNΦ retained the ability to induce neurite outgrowth when physically separated from RGC across a transwell (Fig. 2b, right panel). This suggests that their pro-regenerative effects are, at least partially, dependent on the release of soluble factors. IGF-1 and HB-EGF proteins were upregulated in conditioned media and lysates of the IL-4/G-CSF polarized cells, respectively (Fig. 4a,b). The enhanced neurite outgrowth exhibited by explanted RGC upon co-culture with IL-4/G-CSF polarized BMNΦ, was undermined by exposure of the cultured cells to an αIGF-1 neutralizing monoclonal antibody together with PD153, a small molecule antagonist of epidermal growth factor receptor (EGFR, the cognate receptor of HB-EGF) (Fig. 4c,d). Similarly, co-injection of αIGF-1 and PD153, into the vitreous of mice with ONC injury, partially suppressed the RGC rescue and RGC axon regeneration mediated by adoptively transferred IL-4/G-CSF polarized BMNΦ (Fig. 4e–h). Administration of either αIGF-1 or PD153 alone had no impact on BMNΦ-driven RGC axon regrowth, either *in vitro* or *in vivo* (data not shown). This may be due to the well documented overlap and crosstalk between IGF-1 receptor and EGFR signaling pathways. In fact, many of the biological effects of IGF-1, including stimulation of retinal cell proliferation, are dependent on transactivation of the EGFR^23^.

### IL-4/G-CSF polarized BMNΦ enhance DRG axon regrowth both in vivo and in vitro.

**Figure 5:**
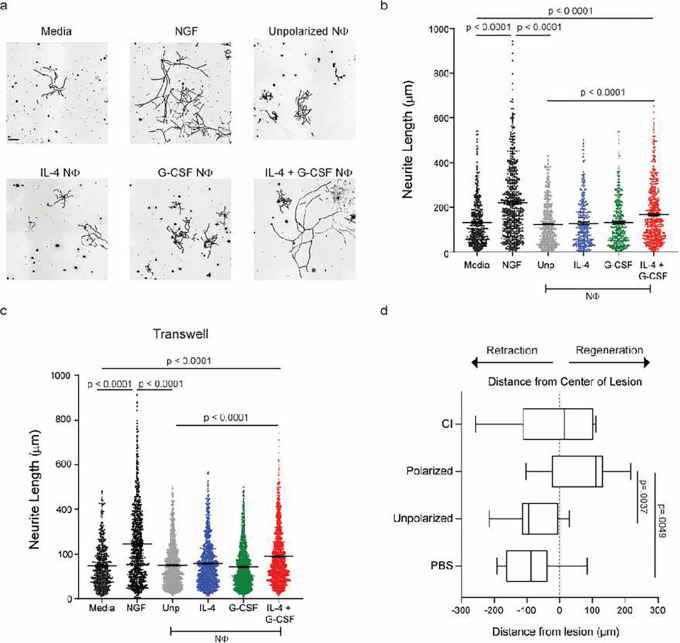
IL-4/G-CSF polarized neutrophils drive the regeneration of spinal cord axons. Ly6G+ BMNΦ, polarized under the indicated conditions, were harvested, washed, and co-cultured with primary DRG neurons for 24 hours. Other DRG neurons were cultured with NGF as a positive control, or in media alone as a negative control. a, Representative images of DRGs from each group. Scale bar, 100 μm. b, Mean length of the longest neurite grown by the cultured DRG neurons. Each symbol represents one neuron. (n=4 mouse per condition, >400 DRGs per condition). Data shown are from one experiment, representative of three independent experiments. c, Mean length of the longest neurite grown by DRG neurons, co-cultured with BMNΦ across a transwell (n=3 mice per condition, >400 DRGs per condition). Data shown are from one experiment, representative of two independent experiments. d, Spinal cord (SC) axon regeneration in mice following transection of SC dorsal columns and administration of various treatments, as indicated. In one group, mice were injected in the right sciatic nerve with IL-4/G-CSF polarized BMNΦ and in the left sciatic nerve with unpolarized BM NΦ on the day of injury. For an additional negative control, other mice were injected in both sciatic nerves with PBS on the day of SC transection. An independent group of mice was subjected to a conditioning injury via sciatic nerve crush 5 days prior to SC transection, as a positive control. Eight weeks following spinal cord transection, all mice were injected with Texas red-conjugated dextran 3,000 Da MW, in the right sciatic nerve, and Alexa Fluor 680-conjugated 3,000 Da MW in the left sciatic nerve. Spinal cords were harvested 10 days later, cleared and imaged. The data show the distance between the lesion center and the rostral tip of the longest regenerating axon (n=8–10 SCs per group). The vertical line in each box plot represents the median, the box indicates the interquartile range, and the whiskers indicate the minimum and maximum values. Data are shown from one experiment, representative of two independent experiments. b-d, Statistical significance was determined by one-way ANOVA followed by Dunnett’s post hoc test

We questioned whether the pro-regenerative effects of IL-4/G-CSF polarized BMNΦ would translate to neuronal subsets other than RGC. Indeed, they readily induced neurite outgrowth of primary dorsal root ganglion (DRG) cells during direct co-culture, or culture across a transwell (Fig. 5a–c). Next, we injected IL-4/G-CSF polarized BMNΦ into the right sciatic nerve of mice that were subsequently subjected to spinal cord injury (SCI) via laceration of the dorsal columns at the T4 level. For an internal negative control, the contralateral (i.e. left) sciatic nerve was injected with unpolarized BMNΦ derived from the same donor pool. For a positive control, an independent group of mice were subjected to conditioning injury (CI) of the sciatic nerves 5 days prior to SCI, a standard method used to trigger axon regeneration in the ipsilateral spinal cord dorsal column^24^. Spinal cord dorsal column axons were traced on day 56 post injury by injection of the sciatic nerves with fluorochrome-conjugated 3000 Da MW dextran. The distance between the lesion center and the most rostral tip of regenerating axons was measured 10 days afterwards. Intraneural (i.n.) injection of IL-4/G-CSF polarized BMNΦ triggered significant axon regeneration in the ipsilateral dorsal column, comparable to that induced by CI (Fig. 5d). In contrast, axons in dorsal columns ipsilateral to sciatic nerves injected either with unpolarized BMNΦ or PBS, had retracted to a level caudal to the lesion center.

### IL-4/G-CSF polarized human BMNΦ induce axon regrowth.

**Figure 6:**
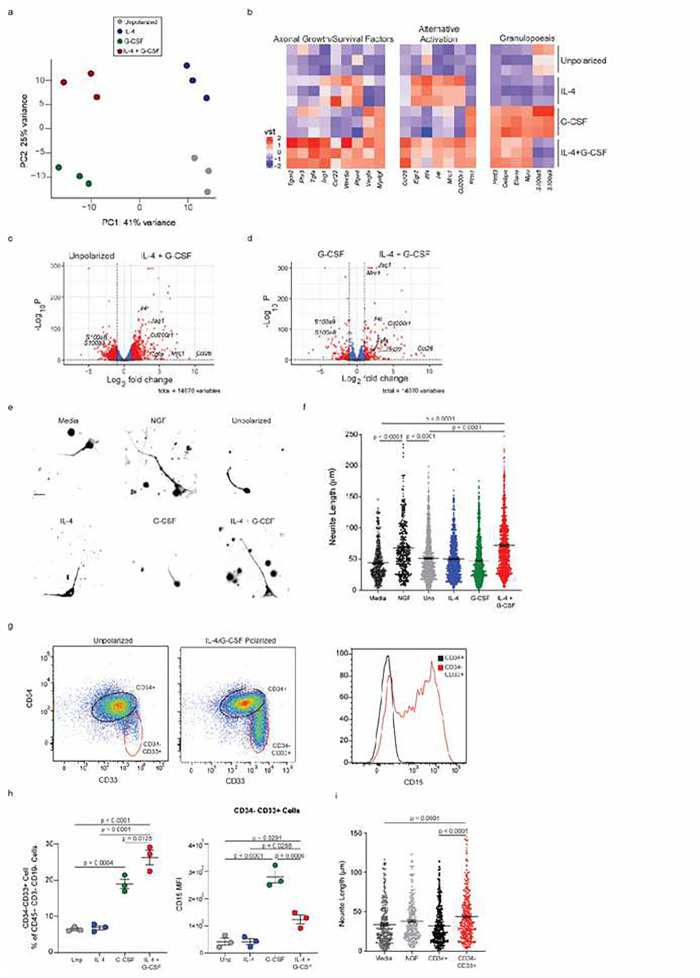
IL-4/G-CSF polarized CD34+ BM cells contain a subset of neuro-regenerative immature neutrophils. CD34+ human BM cells were cultured in media alone, or in the presence of recombinant human IL-4 and /or G-CSF, for 48 hours. a-d, RNA was extracted from cells in each group for bulk RNA sequencing. a, PCA plot. Each symbol represents 1 biological replicate, each comprised of cells derived from a single unique donor (n=3). b, Heat maps depicting scaled expression of selected genes associated with neuroprotection/ axonal regeneration (left), alternative activation (middle), and granulopoesis (right). c, d, Volcano plots illustrating differences in gene expression comparing unpolarized and IL-4/G-CSF polarized human BM cells (c), and G-CSF polarized and IL-4/G-CSF polarized human BM cells (d). Red dots represent genes that are below an adjusted P value of 0.05 and greater than an absolute log2-fold change of 0.25. Green dots show genes that meet the fold change threshold but not the P value threshold. Blue dots show genes that meet the p value threshold, but not the fold change threshold. e, f Human cortical neurons were co-cultured for 24 hours with polarized or unpolarized human CD34+ BM cells. BM cells derived from unique donors were cultured independently of one another. Additional neurons were cultured with recombinant NGF as a positive control, or in media alone as a negative control. e, Representative images of cortical neurons cultured with BM cells from each group, or with NGF or media alone. Scale bar, 20 μm. f, Mean length of the longest neurite grown by the cultured human cortical neurons. Each symbol represents one neuron. (n= 6 unique donors per set of conditions; > 150 neurons per condition). g-h, IL-4/G-CSF polarized or unpolarized CD34+ human BM cells were analyzed by flow cytometry. g, Representative pseudo-color plots showing expression of CD34 and CD33, gated on viable CD45+CD3− CD19− cells (left and middle panels). Histogram showing CD15 expression, gated on CD34+ cells (black) or CD34− CD33+ cells (red), among the IL-4/G-CSF polarized human BM cells (right panel). h, Percentage of CD45+CD19− CD3− cells that are CD33+ in each group (left panel). CD15 MFI, gating on CD33+ cells (right) (n=3 unique donors). Data shown are from one experiment, representative of two independent experiments. i, Human cortical neurons were co-cultured for 24 hours with either CD34+ or CD34− CD33+ cells, which were FACS sorted from IL-4/G-CSF polarized human BM cells. Additional neurons were cultured with recombinant NGF as a positive control, or in media alone as a negative control. Data are shown as the mean length of the longest neurite grown by the cultured human cortical neurons. Each symbol represents one neuron. (n=2 unique donors, >200 neurons per condition). Data are from one experiment, representative of two independent experiments. f, h, i, Statistical significance was determined by one-way ANOVA followed by Dunnett’s post hoc test

Extrapolating from our animal model experiments, we attempted to generate alternatively activated, pro-regenerative *human* myeloid cells by culturing human CD34^+^ BM hematopoietic stem cells, isolated from unique donors, with recombinant human IL-4 and G-CSF. In parallel, BM cells from each donor were cultured without recombinant cytokines, or with either IL-4 or G-CSF individually, as controls. After 48 hours, RNA was extracted from BM cells in each experimental group and subjected to bulk RNA sequencing. The IL-4/G-CSF polarized human BM cells readily separated from unpolarized and single cytokine polarized cells on a PCA plot (Fig. 6a). They expressed a distinctive transcriptomic signature indicative of alternative activation, reflected by differential expression of *IL-4R*, *Mrc1*, *Cd200r1* and *CCL26* (Fig. 6b–d). IL-4/G-CSF polarized human BM cells also expressed elevated levels of genes that have been associated with healing responses, including transforming factor-α (*Tgfa*, a ligand of the human EGFR), Jagged-1 (*Jag1*), and Pentraxin-3 (*Ptx3*). All of these molecules have previously been shown to enhance neurite/axon regrowth and growth cone elongation^25,26^, and to participate in neurorepair pathways^27–29^. The double cytokine polarized human BM cells expressed high levels of genes specific for the neutrophil lineage such as neutrophil elastase and myeloperoxidase, but relatively low levels of the neutrophil maturation markers, *S100a8* and *S100a9* (Fig. 6b–d).

Human BM cells polarized with IL-4 and G-CSF, but not their unpolarized or single cytokine polarized counterparts, significantly enhanced neurite outgrowth of primary human cortical neurons during co-culture (Fig. 6e, f). Flow cytometric analysis revealed that IL-4/G-CSF polarized human BM cells are composed of two populations: a **CD34**^**+**^CD33^−^CD15^−^ subset (consistent with undifferentiated hematopoietic stem cells), and a **CD15**^**+**^**CD33**^**+**^CD34^−^ subset (consistent with immature neutrophils) (Fig. 6g, h). Both subsets are CD3^−^CD19^−^CD56^−^CD14^−^CD16^−^ (data not shown). The classification of the CD15^+^CD33^+^CD34^−^ subset as neutrophils is supported by their expression of neutrophil elastase (*Elane*) and myeloperoxidase (*Mpo*) (Fig. 6b). The fact that those cells express CD33 (which is progressively downregulated during granulopoiesis^30^), and do not express CD10 (data not shown), indicates that they are immature. FACS isolated, IL-4/G-CSF polarized CD15^+^CD33^+^ cells, but not CD34^+^CD33^−^ cells, independently derived from six out of six individual donors, consistently stimulated human cortical neuron neurite outgrowth (Fig. 6i and Extended Data Fig. 4).

## Discussion

In this paper we describe novel populations of BM derived myeloid cells possessing neuroprotective and pro-regenerative properties. These cells were generated *in vitro* via short term culture of either purified murine Ly6G^+^ BM cells, or human CD34^+^ BM stem cells, with a combination of recombinant IL-4 and G-CSF. Notably, our findings suggest that fundamental characteristics of IL-4/ G-CSF polarized neuro-regenerative myeloid cells are conserved across species. Whether of mouse or human origin, these polarized cells, responsible for triggering axon regrowth, have a cell surface phenotype and transcriptomic signature consistent with immature, alternatively activated neutrophils. Both the mouse and human myeloid subsets express EGFR ligands (HB-EGF for mice and TGFα for human), along with a panoply of other neuroprotective agents and growth factors, that could contribute to their reparative functions.

Immature neutrophils expressing markers of alternative activation have also been detected in animal models of myocardial infarction and stroke. The accumulation of atypical neutrophils in the ischemic heart or brain is associated with reduced tissue damage^11,31^. The factors that drive their differentiation *in vivo,* including potential roles of endogenous IL-4 and/ or G-CSF, and their mechanisms of action in mitigating tissue injury, remain poorly understood. Similarly, granulocytic myeloid derived suppressor cells (G-MDCSs), which accumulate in gliomas as well as non-CNS tumors, exhibit characteristics of immature, alternatively activated neutrophils. G-MDSCs produce growth and angiogenic factors that, in conjunction with their potent immunosuppressive properties, enable tumor progression^32^. Tumor associated, TGFβ-dependent “N2” neutrophils bear ring-shaped nuclei and express relatively high levels of *Arg1* and *Vegf* transcripts. N2 neutrophils have been shown to promote tumor growth in several syngeneic mouse models^33^. The extent to which these subsets of alternatively activated neutrophils, emerging in different microenvironments and disease states, share overlapping phenotypes and functions with the murine neuro-regenerative subset described in this paper, remains a subject of active investigation. Further research is warranted to understand the distinctions and similarities between these populations and their potential roles across different neuropathological conditions.

A comparison of murine IL-4/G-CSF polarized BMNΦ and zymosan-modulated, pro-regenerative neutrophils from our previous publications reveals several shared characteristics, but also distinct differences in their properties^12,13^. Both cell populations express neutrophil-specific markers, including Ly6G, G-CSF receptor, myeloperoxidase and neutrophil elastase, along with canonical markers of alternative activation such as Arg1, Mrc1 and IL-4Rα. Additionally, they both display an immature phenotype as indicated by ring shaped nuclei and low levels of cell surface Ly6G and CD101. IL-4/G-CSF polarized murine BMNΦ express F4/80, a marker traditionally associated with the monocyte/macrophage lineage, while the zymosan-modulated cells express CD14, another standard monocyte marker. Interestingly, despite some overlap in transcriptomic and cell surface signatures, nuclear morphology, growth factor profiles, and mechanisms of action, the two populations are not identical^12^. IL-4-G-CSF polarized BMNΦ and zymosan-modulated neutrophils are similar in that both secrete IGF-1, which contributes to their respective neuro-reparative effects. However, optimal RGC axon regeneration induced by zymosan modulated neutrophils requires co-production of NGF^13^, which is not produced by IL-4/G-CSF polarized BMNΦ. Instead, the latter population produces HB-EGF, a factor that is not expressed by their zymosan-modulated counterparts^12^.

The emerging literature indicates that atypical neutrophils with neuro-reparative potential encompass a spectrum of phenotypes. In the peripheral nervous system, distinct neutrophil subsets have been associated with repair processes. For example, Ly6G^+^ SiglecF^+^ neutrophils expressing “neurosupportive” genes (*Efna5* and *Mtap1b*) accumulate in the olfactory neuroepithelium during recovery from intranasal methimazole-induced rhinitis, coinciding with active neurogenesis^34^. Similar to IL-4/G-CSF polarized BMNΦ, these SiglecF^+^ neutrophils express F4/80. However, they have a lobulated nucleus and express relatively high levels of CXCR2. In a separate model, the regeneration of severed sensory axons in the sciatic nerve was accelerated by administration of indole-3-proprionic acid (IPA).^35^ Notably, IPA-enhanced sciatic nerve regrowth relied on the chemotaxis of neutrophils to the dorsal root ganglia via a CXCR2 dependent pathway. In contrast, zymosan-modulated, pro-regenerative neutrophils access the eye by a CXCR2 independent pathway. The diverse phenotypic and mechanistic aspects of neuro-reparative neutrophils are likely to become more evident with the growing use of single cell analytical techniques, enabling more granular investigations of the role of the immune system in limiting and restoring nervous system damage across animal models and human specimens.

We found that IL-4/G-CSF polarized BMNΦ stimulate explanted neurons to grow neurites, in part, by an IGF-1R/ EGFR dependent pathway. Activation of the phosphatidylinositol-3 kinase (PI3K) signaling pathway through IGF-1R has previously been shown to prevent secondary neuronal death after axotomy or other toxic insults, and to drive axon regeneration^36–39^. EGFR levels are upregulated on RGC and DRG in response to trauma^40,41^. Similar to IGF1 signaling, intraneuronal EGFR signaling activates the PI3K pathway^42^, leading to enhanced neuronal survival and neurite outgrowth^43^. Thus, IGF-1 and HB-EGF, secreted by adoptively transferred IL-4/G-CSF polarized BMNΦ, likely engage their respective receptors on neurons in our CNS traumatic injury models, thereby bolstering cell survival and/ or axon regeneration. It is also possible that these growth factors mediate therapeutic effects via modulation of glial cells. For instance, in a mouse model of cerebral hemorrhage, IGF-1 was shown to shift microglial polarization from a neurotoxic to an alternatively activated state^44^. Additionally, EGFR activation by TGFα directly induced astrocytes to acquire a phenotype that supported axon regeneration^45^. In an independent study, EGF-stimulated astrocytes promoted neurite outgrowth from retinal explants^46^. Conditioned media harvested from cultures of primary rat cortical astrocytes, previously activated by an EGF-containing hydrogel, protected neurons against injury and promoted synaptic plasticity^47^. Activation of the EGFR pathway causes astrocytes to produce transforming growth factor β_1_, brain-derived neurotrophic factor, neuritin, platelet derived growth factor α, and fibroblast growth factor 2, each of which could benefit neurorepair^48^. In preliminary studies, that go beyond the scope of the current paper, we found that i.o. injection of IL-4/G-CSF polarized BMNΦ in mice with ONC injury alters gene expression profiles of retinal Muller cells and ON astrocytes, in a manner that might be conducive to healing (data not shown). Collectively, these findings suggest that IGF-1 and HB-EGF may exert their repair-promoting effects on various cellular targets and through multiple mechanisms in the retina and other CNS regions.

The current paucity of therapies capable of actively reversing damage in the CNS highlights the importance of research developments like those presented here. Since human BM stem cells can be safely obtained from living subjects, the findings in this paper offer hope for the development of autologous myeloid-based therapies aimed at restoring lost neurological functions. The potential applications for this approach are vast, ranging from traumatic injury of the optic nerve, spinal cord or brain, to neurodegenerative conditions like multiple sclerosis and ALS. By harnessing autologous myeloid cells derived from the patient’s own BM, this cell therapy could be personalized, making it compatible with the individual’s immune system, and potentially increasing its efficacy.

Various approaches have been explored in animal models to overcome intrinsic and extrinsic barriers to axon regeneration, with varying degrees of success. These approaches encompass genetic reprogramming of neurons, biomaterial transplantation, and the blockade of inhibitory Nogo receptors.^49–51^ Despite promising results from some of these interventions, none have demonstrated curative effects. To achieve positive outcomes in the real world clinical setting, it is likely that a combination of approaches will be necessary, integrating non-redundant and complementary treatments tailored to individual patients. The success of such a strategy hinges on having a diverse armamentarium of synergistic pro-regenerative/ cytoprotective agents at our disposal. Given the distinctive mechanism of action of IL-4/G-CSF polarized BM myeloid cells, they hold significant promise as a potent component within multimodal therapeutic regimens aimed at promoting neuroprotection and repair. Combining these cells with approaches that lower intrinsic or extrinsic barriers to axon regeneration may lead to effective treatments for a range of neurological conditions. Furthermore, ongoing research may lead to the development of therapeutic cocktails consisting of pharmaceutical agents, nanoparticle based therapies, and/ or gene therapies, that replicate the mechanism of action demonstrated by IL-4/G-CSF polarized myeloid cells.

In summary, the findings reported in this paper hold significant implications for the future of regenerative medicine in neurology. The possibility of developing autologous myeloid cell-based therapies, personalized treatment strategies, and innovative therapeutic cocktails, could ultimately bring us close to effective solutions for reversing CNS damage.

## Methods

### Mice.

C57BL/6 wild type (WT) male mice, aged 8–12 weeks, were purchased from Jackson Laboratory. Mice were housed in micro-isolator cages with *ab libitum* access to food and water under a 12 hour light/dark cycle. All animal and surgical procedures were performed in compliance with the Ohio State University Institutional Animal Care and Use Committee.

### Optic nerve crush (ONC) surgery.

Mice were anesthetized with 100 mg per kg body weight (mg kg^−1^) of ketamine and 10 mg kg^−1^ xylazine, via intraperitoneal (i.p.) injection. Optic nerves were exposed under visualization with a Nikon stereomicroscope, and compressed 1–2 mm behind the eye for five seconds using self-close forceps (Dumont no 7, Roboz). Following the procedure, eyes were rinsed with sterile PBS and treated with ophthalmic ointment (Puralube; Fera Pharmaceuticals) to prevent drying. Mice received a subcutaneous injection of buprenorphine extended release at a concentration of 1 mg kg^−1^ immediately prior to surgery. All mice were closely monitored on a daily basis until the endpoint.

### Neutrophil isolation and polarization.

Naïve adult C57BL/6 wild type mice were euthanized via CO_2_ fixation. Mouse femurs, tibias, and humeri were dissected and stripped of muscle. Bone marrow cells were then flushed out with MACS buffer (1X PBS with 0.5% bovine serum albumin (BSA) and 2mM EDTA), using a 30 ml syringe connected to a 25 gauge needle. Neutrophils were isolated from total flushed bone marrow cells via magnetic-activated cell sorting (MACS) with Ly6G magnetic beads (MiltenyiBiotec), following the manufacturer’s instructions. A purity of 95–99% was routinely confirmed by flow cytometry. Isolated neutrophils were counted and resuspended in complete RPMI media (Gibco) containing 10% FBS (Atlanta Biologicals) at a concentration of 1×10^6^ cells per mL. For polarization, the neutrophils were cultured in the presence of recombinant murine IL-4 at 25 ng/mL (Preprotech) and/ or G-CSF at 25 ng/mL (filgrastim, Amgen), for a period of 24 hours at 37°C and 5% CO_2_.

### Human bone marrow polarization.

Purified human bone marrow derived CD34+ cells (StemCell) were thawed, counted, and resuspended in StemSpan SFEM serum-free media (Stemcell) at 1×10^6^ cells per mL. Cells were cultured with or without recombinant human IL-4 (Preprotech) at 25 ng/mL and/ or G-CSF at 100 ng mL^−1^ (filgrastim, Amgen) for 48 hours at 37°C and 5% CO_2_.

### Intraocular (i.o.) adoptive transfer of cells and growth factor inhibitors.

Following a 24 hour culture under neutral or polarizing conditions, murine Ly6G^low^ BMNΦ were harvested, washed, and re-suspended in sterile PBS at a concentration of 1.5×10^5^ cells per μL. The suspension was then injected into the posterior chamber of the eyes of mice using a Hamilton syringe attached to a 32-gauge needle (2 μL/eye/ dose). The same number of cells were injected twice, on days 0 and 3 post-ONC. In some cases, neutrophils were injected in combination with anti-IGF-1 (Abcam) and/or PD153 (Tocris), or isotype matched control antibody (Sigma), all at a concentration of 1 μg uL^−1^.

### Dorsal spinal cord injury (SCI).

Mice were anesthetized with 100 mg kg^−1^ ketamine and 10 mg kg^−1^ xylazine i.p. The T8 lamina were removed, under a stereomicroscope, using micro-rongeurs. The spinal column was exposed, Roboz McPherson-Vannas Micro Dissecting Spring scissors were inserted 1 mm deep, and a hemisection of the dorsal spinal cord was performed, thereby transecting the axons in the dorsal column. Perma-Hand Black sutures (5–0, Ethicon) were used to close the muscle layers, and coated Vycryl sutures (5–0, Ethicon) were used to close skin incisions.

### Sciatic nerve crush/ conditioning injury.

Five days prior to SCI, some mice received a conditioning injury (CI) to the right sciatic nerve. Mice were anesthetized as previously described. The sciatic nerve was compressed at mid-thigh level for 10 seconds using fine forceps (Dumont no. 5). The surgical wound was closed by clipping the overlying skin.

### Intraneural adoptive transfer.

Polarized or unpolarized BMNΦ were injected into the sciatic nerve using a Hamilton syringe with a 32 gauge needle (1.5×10^5^ cells in 1.5 μL PBS, per nerve) on the day of SCI. Right sciatic nerves received an injection of IL-4/G-CSF polarized cells, while the left sciatic nerve received an injection of unpolarized control cells. As an additional control, some mice were injected intraneurally with PBS alone.

### Primary mouse RGC cultures.

Pups were euthanized at age 4–7 days. Eyes were extracted and dissected under a Nikon stereomicroscope. Extracted retinas were placed in 1 mL of 0.05% trypsin (Gibco), triturated 10 times with a glass Pasteur pipette, and incubated in a water bath at 37°C for 5 min. Trituration was then repeated to a achieve a single cell suspension. Trypsin activity was quenched with complete RPMI culture media. The cell suspensions were centrifuged at 800g, supernatant was removed, and cells were suspended in MACS buffer. RGCs were then isolated using Thy1.2 magnetic beads (Miltenyi Biotec). The purified RGCs were suspended in Neurobasal media (Gibco) supplemented with B-27 (Gibco), glutamine (2 mM; Gibco), and penicillin-streptomycin (100 units per mL; Gibco). RGCs were then cultured in 96 well plates coated with poly-L-lysine (Sigma) and laminin (Millipore), at 1×10^4^ cells per well, for 24 hours at 37°C and 5% CO_2_. Polarized or unpolarized neutrophils (1×10^4^ cells per well), or recombinant CNTF (100 ng mL^−1^), were added to some of the wells. In some cases, neutrophils were separated from RGCs via a transwell insert (5 μm pore size; Corning). Following co-culture, RGCs were fixed with ice-cold 4% paraformaldehyde (PFA) for 30 minutes prior to immunohistochemical staining and imaging.

### Primary mouse DRG cultures.

Lumbar (L4–5) DRG neurons were collected from 8–10 week old WT mice and digested with collagenase type 2 (4 mg mL^−1^; Worthington Biochemical) and lipase (1 mg mL^−1^; Sigma-Aldrich) for 45 minutes at 37°C and 5% CO_2_. DRG neurons were counted, resuspended in DMEM/F12 (Gibco) supplemented with 10% FBS (Atlanta Biologicals), glutamine (2 mM; Gibco), and penicillin-streptomycin (100 units per mL; Gibco), and plated at a concentration of 2×10^3^ DRGs per well on laminin/poly-L-lysine coated 24 well plates. DRG neurons were cultured at 37°C and 5% CO_2_, either alone or in the presence of polarized neutrophils (2×10^4^ cells per well). In some cases the DRGs were separated from neutrophils by a transwell insert (5 μm pore size; Corning). Cells were cultured for 20 hours then fixed with ice-cold 4% PFA for 30 minutes prior to immunohistochemical staining and imaging.

### Primary human cortical neuron culture.

Primary human cortical neurons (ScienCell) were thawed, counted, and resuspended in complete neuronal media (ScienCell) supplemented with 1X neuronal growth supplement (ScienCell) and 1X penicillin-streptomycin (ScienCell). Cells were plated at 1.2×10^4^ cells per well in a 96 well plate coated with poly-L-lysine (Sigma) and laminin (Millipore). The human neurons were cultured, with or without polarized or unpolarized CD34+ human bone marrow cells (StemCell), for 24 hours at 37°C and 5% CO_2_. Following the completion of cultures, neurons were fixed with ice-cold 4% paraformaldehyde (PFA) for 30 minutes prior to immunohistochemical staining and imaging.

### Cytokine quantification.

IGF-1 levels were measured in 24 hour conditioned media of pre-polarized BMNΦ that had been resuspended in fresh serum free neurobasal media at a concentration of 4×10^6^ cells per mL. IGF-1 levels were quantified by enzyme-linked immunosorbent assay (ELISA) (R&D Systems) according to the manufacturer’s instructions. HBEGF levels were quantified in by Western blot analysis. Cells were resuspended in 50 μL RIPA Buffer (Sigma), and briefly sonicated. Total protein in cell lysates was then quantified via Bradford assay (Pierce) according to kit instructions. Equal amounts of protein from each lysate were loaded onto a 10% Gel. The gel was run at 100V for 2 hours. Protein was transferred onto a PVDF (Bio-Rad) membrane at 200 mA for 2 hours at 4°C. The membrane was blocked in 5% BSA for 1 hour, and then stained with anti-HBEGF antibodies (R&D), or anti-β-Actin antibodies (Bio-Rad), overnight at 4°C. Membranes were then stained with donkey anti-sheep HRP antibody (HBEGF, 1:100) or goat anti-mouse HRP secondary antibody (β-Actin, 1:2500). Blots were visualized using SuperSignal West Femto (ThermoFischer) reagent and imaged on an Odyssey XF Imager (LiCor). Blot analysis was performed using Image Lab (Bio-Rad).

### Immunohistochemistry and quantification of regenerating axons, viable RGCs, and neurite length

#### Optic nerves

Two days prior to euthanasia, mice received an i.o. injection of Alexa Fluor 647 conjugated Cholera Toxin B (CTB; Invitrogen) at 5 μg in 2 μL PBS per eye. Euthanized mice were perfused with PBS. Optic nerves were dissected and fixed for 2 hours in 4% PFA at room temperature. Post fixation, nerves were dehydrated in 100% methanol for 4 minutes, then placed in Visikol Histo-1 solution (Visikol) overnight at 4°C. Nerves were then transferred to Visikol Histo-2 solution (Visikol) for 2 hours and imaged in Visikol Histo-2 solution. Whole nerves were imaged using an Olympus IX83 inverted confocal microscope. Images were processed using ImageJ software, generating 6 μM slices. Regenerating CTB+ axons were counted at 200 μm intervals past the injury site, up to 1600 μm, utilizing a superimposed grid (3 sections per nerve were averaged). The number of labeled axons per section were normalized to the width of the section and converted to the total number of regenerating axons per optic nerve as described previously^6^. All counting was performed in a blinded fashion.

#### Retinas

For whole mount analysis, retinas were dissected under a stereomicroscope. Retinas were fixed in 4% PFA for 2 hours at room temperature, after which they were stored in 1X PBS overnight at 4°C. Retinas were washed three times in 1X PBS with 3% Triton X100 for 1 hour, incubated with blocking buffer (1X PBS with 5% normal goat serum and 3% Triton X100) for 2 hours, then incubated with primary antibodies (rabbit anti-mouse Brn3a, Synaptic Systems) at 4°C for 72 hours. Retinas were washed and incubated with secondary antibodies (goat anti-rabbit 59, Invitrogen) at 4°C for 24 hours. The stained retinas were mounted onto charged slides and imaged using an inverted Olympus IX83 fluorescent microscope at 40X. Brn3a+ RGCs were counted over eight fields distributed between four quadrants per retina, and quantified using Imaris V9.8 imaging software.

For cross-sectional images of the retina, eyes were dissected, post-fixed as described above, embedded in Tissue-Tek OCT compound (Sakura Finteck) and stored at −80°C. Sections (25-μm) were cut with a cryostat and mounted on Superfrost Plus microscope slides, rinsed with PBS, blocked with 5% normal goat serum in PBS with 0.25% Triton X-100 (PBS-T) at 25 °C, and incubated with primary antibodies (anti-GFAP at a 1:500 dilution in PBS-T + 5% goat serum) at 4 °C overnight. The next day, sections were washed with PBS-T and incubated with secondary antibodies (goat anti-mouse Alexa Fluor 488, Invitrogen) in blocking solution (PBS + 5% goat serum) at 25 °C for 2 h. DAPI (300 nM) was used to counterstain sections.

#### Spinal cords

Eight weeks following spinal cord dorsal column laceration, mice were injected with Texas red-conjugated dextran 3,000 Da MW tracer (microruby, Life Technology; 1.5 μl of 10% solution) in the right sciatic nerve, and Alexa Fluor 680-conjugated 3,000 Da MW tracer (microruby, Life Technology; 1.5 μl of 10% solution) in the left sciatic nerve using a Nanofil 10-μl syringe with a 36 gauge beveled needle (World Precision Instrument). Ten days later, mice were euthanized and perfused with PBS. The spinal cord was dissected, and dehydrated overnight in 50% ETOH at 4°C. The spinal cords were then transferred to a 70% ETOH solution for 24 hours, followed by a third dehydration step at 100% ETOH for an additional 24 hours. Spinal cords were washed once with 100% ETOH for 4 hours before being placed in Visikol Histo-1 for 24 hours at 4°C. Whole spinal cords were then placed in Visikol Histo-2 and imaged using an Olympus IX83 inverted confocal microscope. The length between the most rostral tip of the labeled axons and the epicenter of the lesion site was measured using Imaris V9.8.

#### Murine RGC and DRG, and human cortical neurons

Plates were washed in PBS with 0.1% TritonX 100 (PBS-T) prior to blocking with 10% goat serum in PBS-T for 1 hour at room temperature. After two washes in PBS-T, cultured neurons were incubated with anti-βIII tubulin (TUJ1, Promega) in 3% BSA in PBS-T overnight at 4 °C. Following two washes, the samples were incubated with Alexa Fluor 488-conjugated secondary antibody (Invitrogen) for 2 hours at room temperature. Samples were washed twice with PBS and left in a solution of PBS for imaging. RGCs and human cortical neurons were imaged at 20X and DRG neurons at 10X, using an Olympus IX83 inverted fluorescent microscope. Images were analyzed with Neuromath (Weizmann Institute of Science) to identify the longest neurite.

### Cytospins

Purified and polarized neutrophils were subjected to cytospin for 5 minutes at 5×10^3^ rpm. Slides were air dried and stained with Wright-Giemsa solution (Abcam) and imaged using an Olympus SZH Zoom Stereo Microscope to visualize nuclear morphology.

### Flow cytometry

Flow cytometry was performed as previously described^13^. Briefly, mice with euthanized via isoflurane overdose. Blood was collected and place in a tube coated with EDTA (Sarstedt Inc). Lysis of red blood cells was performed using Ammonium-Chloride Potassium Lysing Buffer (Quality Biological). Bone marrow neutrophils were polarized for 24 hours, centrifuged, and re-suspended for staining.

Both human and mouse cells were labeled with fixable viability dye eFluor 780 (eBioscience), and blocked with anti-CD16/32 (clone 2.4G2). Mouse cells were stained with anti-mouse fluorochrome-conjugated antibodies specific for CD45 (30-F11), CD11b (clone M1/70), Ly6G (1A8), F4/80 (T45–2342), CD14 (rmC5–3), CD115 (T38–320), and CD124 (mIL4r-M1), all from BD Biosciences; and CD101 (polyclonal) from eBioscience. Human cells were stained with anti-human antibodies specific for CD3 (HIT3a), CD10 (HI10a), CD14 (MφP9), CD15 (W6D3), CD16 (19.2), CD19 (SJ25C1), CD33 (HIM3–4), CD34 (581), and CD45 (HI30), all purchased from BD Biosciences. For intracellular staining, cells were fixed with Cytofix/Cytoperm buffer (BD Biosciences), permeabilized with 1X Perm wash (BD Biosciences), and stained with fluorescent antibodies specific for CD206 (MR6F3; eBioscience). Flow cytometry was performed on a FACS Symphony A3 cell analyzer (BD Biosciences); flow sorting was carried out on a FACS Melody cell sorter (BD Biosciences). Cells were gated using forward and side scatter after doublet exclusion and analyzed on FlowJo v10 software.

Using FlowJo v10 software, t-distributed stochastic neighbor embedding (t-SNE) plots were created through downsampling of each group to 20,000 total cells. Samples were concatenated and t-SNE was calculated with a perplexity of 80 at 10,000 iterations and a default learning rate.

### Quantitative real time PCR

Cells were centrifuged and resuspended in 300 μL of RLT Buffer (Qiagen). RNA was purified using Qiagen RNeasy Mini Kit per manufacturers instructions. RNA was converted to cDNA using a high capacity cDNA reverse transcription kit (Life Technologies)., PowerUP SYBR Green Master Mix (Applied Biosystems) was used to perform RT-qPCR using a Quant Studio Design v1.3 (Applied Biosystems). Analysis was done using the ΔΔCt method, with unpolarized cells as control. Values were normalized relative to *Actb*.

Primers:

*Actb*: FP: 5’-CAGGTATCCTGACCCTGAAGT-3’

RP: 5’-CACACGCAGCTCATTGTAGA-3’

*Arg1*: FP: 5′-CTCCAAGCCAAAGTCCTTAGAG-3′

RP: 5′-AGGAGCTGTCATTAGGGACATC-3′

*Il4ra*ː FP: 5′-ACACTACAGGCTGATGTTCTTCG-3′

RP: 5′-TGGACCGGCCTATTCATTTCC-3′

*Mrc1*: FP: 5′-AAGGCTATCCTGGTGGAAGAA-3′

RP: 5′-AGGGAAGGGTCAGTCTGTGTT-3′

*Adgre1*: FP: 5’- TGACTCACCTTGTGGTCCTAA-3’

RP: 5’-CTTCCCAGAATCCAGTCTTTCC-3’

### Bulk RNA Sequencing

#### RNA Isolation for bulk sequencing

RNA was isolated from MACS sorted mouse Ly6G^+^ bone marrow cells or human CD34+ bone marrow cell,s following culture under the indicated conditions, using the total RNA purification plus kit (Norgen Biotek Corp.-Cat#−48400). RNA quality was determined by high sensitivity RNA screentape assay (Agilent Technologies-Cat# 5067–5579). Only RNA with RIN # >7 was used for subsequent library preparation. Next, rRNA was depleted (200ng total RNA per sample) using the NEBNext rRNA depletion kit v2 (New England BioLabs-Cat#-E7400). RNAseq libraries were prepared following the manufacturer’s standard protocol (NEBNext ultra II directional RNA library prep kit for Illumina-NEB #E7760).

#### Analysis of mouse BMNΦ RNA sequencing data

Individual FASTQ files were trimmed for adapter sequences and filtered using fastp v0.20.0. Mouse reference genome GRCm38.p6 and gene annotation, described by Gene Transfer Format (GTF), were downloaded from Ensembl release 99 (January 2020). Reads alignment was performed against the reference genome using HISAT2 v2.1.0. Gene expression values for genes were quantified using the featureCounts tool of the Subread package v1.5.0-p2 in unstranded mode. Differential expression was performed with DeSeq2^52^. Volcanos were plotted with R package EnhancedVolcano; deseq2 rlog transformation was used for PCA plotting.

#### Analysis of Human CD34+ BM cell RNA sequencing data

Basic analysis were performed using our in-house pipeline^53^. Raw fastq was aligned to human reference genome GRCh38 with hisat2 v2.1.0^54^. Gene wise counts were generated with featureCounts from the subread package v1.5.1^55^. for genes annotated by ensembl Homosapiens.GRCh38.101, counting the primary alignment in the case of multimapped reads. Counts for a sample across lanes were summed and genes with count > 1000 across the dataset were kept for analysis. Differential expression was performed with DeSeq2^52^.In an A vs C comparison, a positive fold change will be higher in C and a negative fold change will be higher in A. Volcanos plotted with R package EnhancedVolcano, deseq2 vst transformation used for PCA and heatmap plotting.

### Statistical analysis

RGC, DRG, cortical neuron frequency, RGC survival, flow cytometry, RT-qPCR, spinal cord axon outgrowth, and ELISA data were compared between groups using one-way ANOVA followed by Dunnett’s post hoc test using GraphPad 9.5 (Prism). An unpaired Student’s t-test was used to compare axon density between two groups.

## Data Availability

Our bulk RNA-seq data that support the findings of this study have been are deposited in NCBI’s Gene Expression Omnibus (GEO) under accession code GSE244934.
